# Extracellular vesicles: A new diagnostic biomarker and targeted drug in osteosarcoma

**DOI:** 10.3389/fimmu.2022.1002742

**Published:** 2022-09-23

**Authors:** Xiaozhuo Gao, Bo Gao, Shenglong Li

**Affiliations:** ^1^ Department of Pathology, Liaoning Cancer Hospital & Institute, Cancer Hospital of Dalian University of Technology, Cancer Hospital of China Medical University, Shenyang, China; ^2^ Department of Bone and Soft Tissue Tumor Surgery, Liaoning Cancer Hospital & Institute, Cancer Hospital of Dalian University of Technology, Cancer Hospital of China Medical University, Shenyang, China

**Keywords:** EVs, osteosarcoma, biomarkers, treatment, diagnosis

## Abstract

Osteosarcoma (OS) is a primary bone cancer that is highly prevalent among adolescents and adults below the age of 20 years. The prognostic outcome of metastatic OS or relapse is extremely poor; thus, developing new diagnostic and therapeutic strategies for treating OS is necessary. Extracellular vesicles (EVs) ranging from 30–150 nm in diameter are commonly produced in different cells and are found in various types of body fluids. EVs are rich in biologically active components like proteins, lipids, and nucleic acids. They also strongly affect pathophysiological processes by modulating the intercellular signaling pathways and the exchange of biomolecules. Many studies have found that EVs influence the occurrence, development, and metastasis of osteosarcoma. The regulation of inflammatory communication pathways by EVs affects OS and other bone-related pathological conditions, such as osteoarthritis and rheumatoid arthritis. In this study, we reviewed the latest findings related to diagnosis, prognosis prediction, and the development of treatment strategies for OS from the perspective of EVs.

## Introduction

Osteosarcoma (OS) predominantly occurs among individuals below 20 years and is a form of aggressive primary bone cancer (1, 2). The etiology of OS is mainly characterized by epidemiological, genetic, and environmental factors (3). Several risk factors are associated with tumorigenesis of OS, such as alkylating agents, hereditary retinoblastoma, Paget’s disease, ionizing radiation, and chromosomal abnormalities (4, 5). The diagnosis of OS relies mainly on clinical manifestations, medical imaging, tissue biopsy, and laboratory tests. The standard treatment regimens for OS include neoadjuvant chemotherapy, surgical resection, chemotherapy, and interventional therapy (6, 7). Recent developments related to the treatment of OS include extensive research on stem cell therapy, immunotherapy, and gene therapy (8–10). However, due to the complexity of therapeutic interventions and the genetic differences between laboratory animals and humans, these strategies are limited to preclinical studies. Additionally, patients with OS have a high incidence of early lung metastasis, except for other bone tissue metastasis. About 18% of OS patients show signs of micrometastasis at the time of diagnosis, and the five-year survival rate of patients with stage III OS or higher stages of OS is very low (11–13). Moreover, the treatment outcomes are suboptimal because of the difficulty in early diagnosis, the early onset of metastasis, and high malignancy (14, 15). The five-year survival of OS patients who do not receive chemotherapy is below 30%. Pulmonary metastasis is the main cause of OS-related mortality. Moreover, the chemotherapeutic intervention can partially control pulmonary metastasis of OS and increase the five-year survival to 50%. For OS cases with pulmonary metastasis, the two-year survival is less than 25%. Additionally, although there are several alternatives, the survival period during treatment might stabilize without any improvement. Therefore, implementing traditional treatment strategies might not yield the best results (16, 17). Hence, determining the mechanism of the occurrence and metastasis of OS might help to find new clinical diagnostic markers and efficient therapeutic targets.

Extracellular vesicles (EVs) are specialized membranous vesicles originating from endonuclear bodies with particles ranging from 30 to 100 nm in diameter (18, 19). EVs were first identified as a component of blood erythrocytes. They appeared as a lipid bilayer structure surrounded by cytoplasm and devoid of any organelles (20). These EVs were discovered approximately 40 years ago (20). The understanding of the role of EVs in human pathophysiological processes has improved significantly.

Several studies have shown that EVs are produced by various cancer and healthy cells (21–23). When EVs were discovered, their primary function was thought to be the excretion of metabolic wastes from cells (24). However, various studies highlighted the ability of EVs to perform cellular communication, which is essential during various biological processes and disease progression. This communication is possible due to the presence of various nucleic acids and proteins that are responsible for distinguishing the transmission of important biological information between cells (25–28). Thus, EVs can be used as nano-cargos for delivering nucleic acids (such as messenger RNA) (29) and therapeutic agents (such as paclitaxel) (30). Cells within the tumor microenvironment (TME) of OS can secrete EVs, which can deliver non-coding RNAs (ncRNAs) and proteins within the tumor matrix essential for cellular communication. Thus, EVs can effectively regulate the TME within OS and accelerate cell proliferation and metastasis. Additionally, EVs show high systemic stability and are not susceptible to cellular enzymes. They also have good therapeutic and diagnostic potential. In this article, we reviewed the different types of EVs and their biological properties, along with their potential in the diagnosis and treatment of OS.

## The sources of EVs involved in osteosarcoma

Extracellular vesicles secreted by drug-resistant cells facilitate and transfer drug resistance to different types of tumors, including breast, prostate, colon, lung, and gastric cancer, as well as, osteosarcoma (31). Doxorubicin and cisplatin resistance are transferred from OS resistant cells to sensitive cells through EVs that carry P-glycoprotein, MDR-1 mRNA, or the circular RNA hsa_circ_103801 [178.179]. Bone marrow-derived mesenchymal stem cell-derived extracellular vesicles (BMSC-EVs) can promote the proliferation, invasion, and migration of osteosarcoma cells *via* the MALAT1/miR-143/NRSN2/Wnt/β-catenin axis (32). Additionally, EVs secreted by the osteosarcoma 143B cell line contain a pro-osteoclastogenic cargo, which includes MMPs (MMP-1 and MMP-13), RANK-L (Receptor Activator of Nuclear Factor κ B Ligand), CD-9, and TGF-β. These findings highlighted that EVs from different sources exhibit different biological activities.

## The characteristics of EVs

Extracellular vesicles released from most cells contain various proteins, RNA, genomic DNA (gDNA), non-coding RNAs (ncRNAs), lipids, and metabolites (33, 34). EVs can be categorized into three types based on their size and release mechanisms and include EVs, microvesicles, and apoptotic vesicles, with vesicle sizes ranging from 30 to 150 nm, 100 to 1,000 nm, and 50 to 1,500 nm, respectively (35, 36). EVs are cultured from OS cells obtained *in vivo* and purified by differential centrifugation. The separated and purified EVs are assessed according to their purity and morphology, followed by protein profiling and sequencing of the components. The assessment of the morphology of EVs by electron microscopy remains a gold standard. Additionally, flow cytometry (FCM) might also be performed for assessing EVs. For particle size analysis of EVs, Nanoparticle Tracking Analysis Technology (NTA) is frequently used. The production of EVs involves the initiation of endocytosis, the formation of multivesicular bodies (MVBs), and the production of exosomes (37, 38). EVs start to develop with the initial formation of plasma membrane invaginations into a cup-like structure containing cell surface proteins, soluble proteins, and endoplasmic reticulum (ER). This cup-shaped structure, together with trans Golgi, promotes the formation of early endonucleosomes (39). Early intranucleosomes mature into late intranucleosomes, resulting in the formation of MVBs. These MVBs may fuse with the plasma membrane to release the intraluminal vesicles (ILVs) associated with EVs or may fuse with autophagosomes or lysosomes for degradation (40, 41). EVs are found in different types of body fluids, such as urine, plasma, breast milk, and ascites (42, 43), which makes EVs a significant tool with great diagnostic potential.

## The process of the formation of EVs

Extracellular vesicles are usually formed by endosomal endocytosis, in contrast to other conventional membrane outgrowth processes, which deform membranes from organelles into the cytoplasm. The endosomal limiting membrane undergoes multiple depressions with inward growth resulting in the formation ILVs. These ILVs are then converted into MVBs, which have a dynamic subcellular architecture. Interestingly, MVB formation can occur at the endosomal limiting membrane by the endosomal sorting complex required for the transport (ESCRT) mechanism (44, 45). The ESCRT machinery functions through a set of cytoplasmic protein complexes by recognizing the ubiquitinylated modified membrane proteins. The first ESCRT complex (ESCRT-0) can recognize ubiquitin markers, showing high levels of enrichment in the endosomal membrane during the transport of ubiquitinated complex into ESCRT I/II. Within ESCRT I, tumor susceptibility gene 101 protein (TSG101) can detect disulfide bonds and induce depression of the endosomal membrane. They function as shears in the bud neck under the influence of ESCRT III and lead to the formation of MVBs (46, 47). However, MVBs can still be formed in the absence of ESCRT. The process is initiated by an accessory protein ALG-2 interacting protein X (AIix). AIix directly binds to the intracellular bridging protein syntenin, which is further involved in EV formation (48, 49). Such ESCRT-independent MVBs are produced under the action of the abundant tetra-transmembrane protein CD63-α on MVBs and by ceramide-mediated cell membrane outgrowth (50, 51). These MVBs can fuse with lysosomes, degrade their contents, and recirculate them. The sorting of MVBs is significantly regulated by their cholesterol levels. For example, MVBs rich in cholesterol are targeted to cell membranes to be released as EVs, whereas, MVBs with low cholesterol levels are targeted for transport toward lysosomes (52).

## Mechanism of action of EVs

Extracellular vesicles are generally responsible for inducing functional responses in receptor cells by delivering their contents, promoting phenotypic changes in receptor cells, and affecting their physiological state (25, 53). EV-mediated intercellular communication within plasma membrane relies on the activation of surface receptors on recipient cells and initiates cell signaling. The uptake of EVs by recipient cells is facilitated by cytokinesis (54, 55). The mechanisms of exosome cell membrane interaction and the transport of exosomes and endosomes are not fully understood. However, some studies have shown that these mechanisms are associated with the origin of EVs, receptor cells, and downstream processes involved in the same. Some studies have shown the activity of EVs derived from certain cells along with their application in the treatment of diseases (56, 57). The interaction between proteins significantly expressed on EVs and surface receptors of the recipient cell membrane can be used to assess the target cell specificity (58, 59). The known mediators of cell communication also include transmembrane tetraspanins, integrins, lipids, and extracellular matrix components (60, 61).

## Extracellular vesicles in tumor diagnosis and treatment

Extracellular vesicles influence the exclusion of redundant and nonfunctional cellular components (62, 63). They can also act as intercellular linkers for protein, nucleic acid, and lipid transport between host and recipient cells. They strongly affect different biological processes, such as antigen presentation, angiogenesis, inflammation, and apoptosis (64–67). These processes might be related to the metastasis of biomolecules and cell crosstalk that leads to cancer-related events (47, 68, 69). The constituent nucleic acids, proteins, and lipids captured by EVs during production might reflect their cellular origin and physiological state.

These biomolecules have high disease specificity and might act as potential biomarkers. Additionally, EVs function as carriers for these biomolecules and prevent their enzymatic degradation. Various tumor-associated events involve EVs for cell proliferation, apoptosis, metastasis, and angiogenesis, and thus, may be used as a noninvasive diagnostic biomarker in various types of cancer (70–72). For example, miR-21, miR-124–3p, and miR-222 in serum EVs might be used as molecular biomarkers for assessing early cancer development during postsurgical management of high-grade gliomas (HGG) (73). Shin et al. reported the expression of miR-21, miR-451, and miR-636 in urinary EVs in prostate cancer patients, which indicated a close resemblance with preoperative prostate-specific antigen (PSA) levels. Thus, urinary exosome-derived miRNAs might be used as noninvasive markers for predicting prostate cancer prognostic outcomes and metastasis (74). Wang et al. showed that plasma exosome-derived miR-363–5p was necessary for differentiating LN-positive breast cancer (BC) patients from LN-negative patients. Additionally, upregulation of miR-363–5p was strongly associated with overall survival (75). Exosome therapeutic research is focused on three main areas, which include biomedicine, drug delivery, and regenerative medicine. EVs are promising for treating disorders due to their nontumorigenic risk and bactericidal infiltration. Due to their small size, EVs can reach the site of injury through internal circulation and lower immunogenicity, which makes them an ideal candidate for developing treatment against various disorders (76, 77). EVs also facilitate gene delivery to recipient cells, thus alteringtheir biological activity. They are also capable of carrying therapeutic payloads such as proteins, RNAs, and chemotherapeutic agents and delivering them to the target site across different biological barriers (47, 78, 79). EVs can be engineered to target cell signaling pathways or specific recipient cells using a ligand-targeted approach (27, 80). Chemotherapeutic loaded EVs can target tumors with a significant reduction in dose-dependent side effects of chemotherapeutic agents and an increase in their efficacy in cancer treatment (55, 68, 81). Mesenchymal stem cell (MSC)-derived EVs can be used in the field of regeneration and repair. Additionally, some *in vitro* and *in vivo* studies have investigated its regenerative potential and therapeutic applications. In some studies, EVs were found to outperform MSCs in the treatment of various diseases (19, 82, 83).

## Role of EVs in tumor growth and metastasis of osteosarcoma (OS)

**Figure 1:**
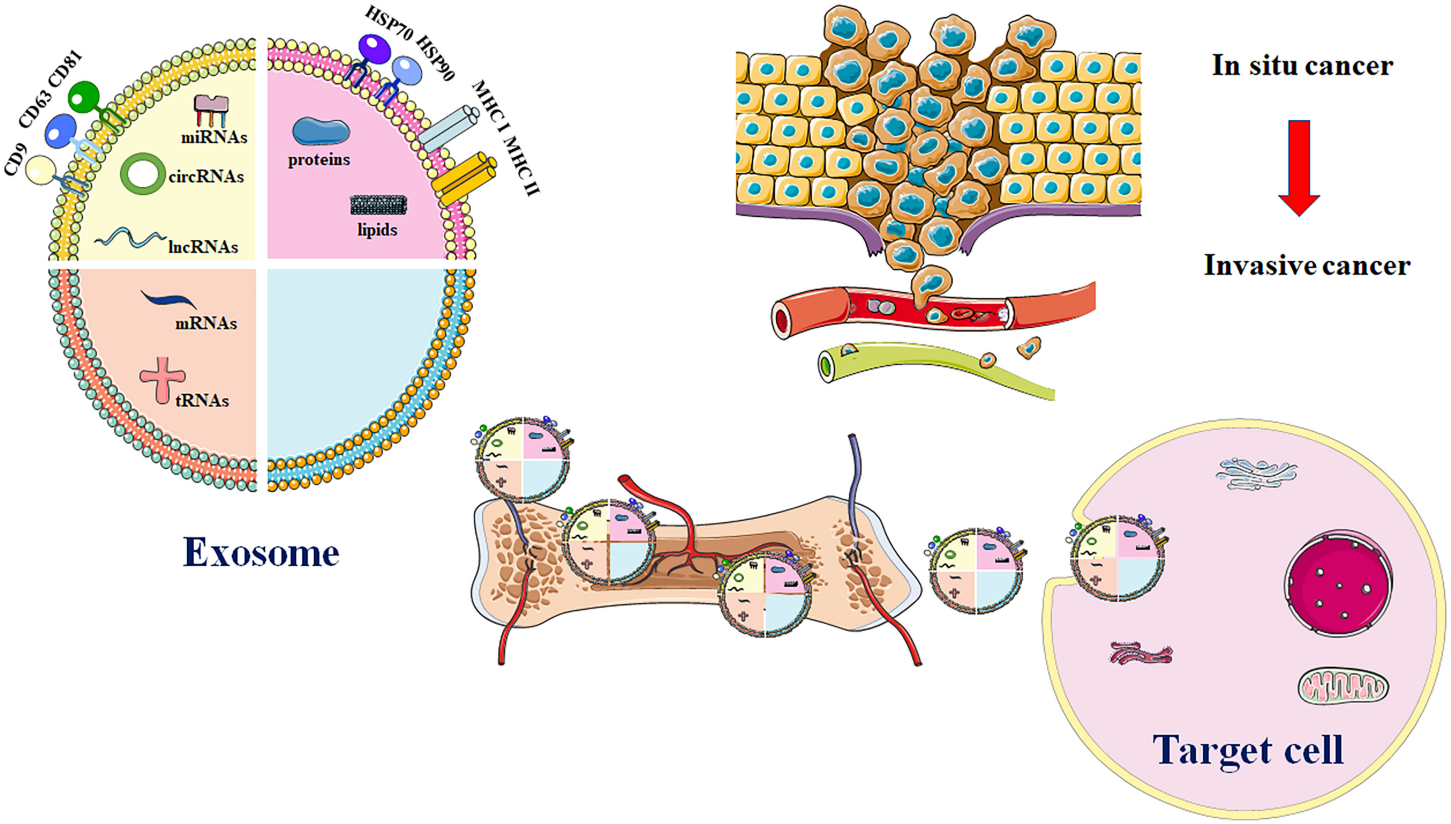
Major exosome release process in OS. EVs are comprised of various proteins and nucleic acids. These evolutionarily conserved proteins that can be used as biomarkers, like HSP70, CD9, CD63, and CD81. Additionally, exosomal cargos are also involved in transport of multiple biomolecules such as DNA or RNA. EVs that carry genetic materials are utilized in development of treatment for OS through enhancing drug resistance, immune evasion, migration, invasion, and angiogenesis. Source cell-derived exosomal cargos are also carried into recipient cells *via* blood circulation. Highly invasive OS cells enhance cell migration and invasion through production of exosomes.

Extracellular vesicles affect cellular communication between cells within the TME, thus influencing cell proliferation and metastasis in cancer. Bone marrow-derived mesenchymal stem cell-derived extracellular vesicles (BMSC-EVs) can promote proliferation, invasion, and migration of osteosarcoma cells *via* the MALAT1/miR-143/NRSN2/Wnt/β-catenin axis (32). This enhancement in cell proliferation and metastasis is facilitated by the epithelial-mesenchymal transition (EMT) in related cell types. Moreover, the TME significantly accelerates tumor neovascularization, immunosuppression through stromal cells, and the transformation of cancer-associated fibroblasts (84–87). In conclusion, EVs have a strong effect on OS cell proliferation, migration, invasion, and angiogenesis by participating in intercellular communication and controlling cellular signaling **(** **Figure 1** **).**

## Extracellular vesicles are involved in osteosarcoma proliferation

Cancer cells undergo indefinite proliferation (88). In contrast, normal tissues have precise and controlled release of pro-growth signals, which cyclically initiate cell proliferation and differentiation up to a finite number of cell divisions. However, tumor cells can inherently produce growth factor receptors, thus escaping negative feedback regulation against proliferation (89, 90). EVs also have an important effect on proliferation in OS **(** **Table 1** **)**. Zhang et al. reported the effect of exosomal miR-208a obtained from bone marrow-derived mesenchymal stem cells (BMSCs) on OS cell proliferation and apoptosis. They found that OS cell growth was enhanced and apoptosis was inhibited when PDCD4 expression was suppressed. This, in turn, activated the Hippo and ERK1/2 pathways. In contrast, the exosomal miR-206 obtained from BMSCs suppressed cell growth, invasion, and migration. It also promoted apoptosis by targeting TRA2B in OS cells (102). Additionally, BMSC-derived EVs could encapsulate and translocate PVT1 in OS cells, and PVT1 promoted cancer development and migration by binding to miR-183–5p and facilitating the expression of ERG (94). BMSC-EVs could enhance OS cell growth, migration, and invasion through MALAT1/miR-143/NRSN2/Wnt/β-catenin signaling (93). Huang et al. showed the effect of EVs obtained from hBMSCs on tumorigenesis and migration. The EVs showed enhanced tumorigenesis and migration by promoting oncogenic autophagy in OS (95). EVs derived from ADSC could enhance OS cell growth, invasion, and migration by delivering COLGALT2 to OS cells, leading to the malignant progression of OS (96). Li et al. found that OS cells that showed AXL upregulation promoted the secretion of EVs into cells with downregulated AXL, and this promoted cell growth, invasion, and migration *via* the linc00852/miR-7–5p/AXL regulatory axis (103). Ge et al. found that BMSC-derived EVs translocate into OS cells and promote OS growth and migration by LCP1/JAK2/STAT3 signaling and inhibit OS progression *via* miR-135a-5p/LCP1 signaling (98). The MG-63 cell-derived EVs, which were co-cultured using HOS and MG-63 cell lines, significantly enhanced OS cell growth and inhibited apoptosis. This effect might be related to the interaction of Hic-5 with smad4 and a decrease in the expression of TCF/LEF that regulates Wnt/β-catenin signaling (99). Han et al. found that exosomal miR-1307 obtained from OS cells can promote OS cell growth, invasion, and migration by inhibiting AGAP1 expression. This finding indicated that the miR-1307-AGAP1 axis might act as an anti-OS therapeutic target (100). Wu et al. found that exosomal miR-15a expression decreased in plasma EVs, and exosomal miR-15a was absorbed by OS cells, which suppressed GATA2/MDM2 signaling *via* the p53 pathway. This inhibited OS cell growth and migration *in vitro* (104).

**Table 1:** Biological activity of exosomes in OS proliferation.

EV content	Parent cells	Target cells	Mechanism	Biological activity	Ref.
miR-208	BMSCs	OS cells	PDCD4/ERK1/2	Enhance OS cell invasion, viability as well as clone formation ability	(91)
miR-206	BMSCs	OS cells	TRA2B	Suppress OS cell growth, invasion, and migration, while inducing their apoptosis	(92)
MALAT1	BMSCs	OS cells	MALAT1/miR-143/NRSN2/Wnt/β-catenin	Promote OS cell proliferation, metastasis, and invasion	(93)
PVT1	BMSCs	OS cells	PVT1/miR-183–5p/ERG	Promote OS proliferation and invasion	(94)
ATG5	BMSCs	OS cells	/	Enhance OS cell growth, invasion, and migration,	(95)
COLGALT2	ADSCs	OS cells	/	Enhance OS cell growth, invasion, and migration	(96)
Linc00852	high AXL expression in OS cells	low AXL expression in OS cells	Linc00852/miR-7–5p/AXL	Promote cell proliferation, migration and invasion	(97)
LCP1	BMSCs	OS cells	miR-135a-5p/LCP1/JAK2/STAT3	Enhance OS cell growth, and migration	(98)
Hic-5	MG-63	MG-63 and HOS cells	Hic-5/smad4-TCF/LEF-Wnt/β-catenin	Promote cell proliferation and inhibit cell apoptosis	(99)
miR-1307	OS cells	OS cells	AGAP1	Enhance OS cell growth, invasion, and migration	(100)
miR-15a	Serum-derived exosome	OS cells	miR-15a/p5/GATA2/MDM2	Inhibit OS cell growth, invasion, and migration	(101)

## EVs have an important effect on OS metastasis

In epithelial-mesenchymal transition (EMT), the epithelial properties of epithelial cells are lost, while the mesenchymal phenotype is acquired. This phenomenon is widely involved in physiological regulation and pathological changes and is closely related to embryogenesis, tissue regeneration, invasion, and metastasis of cancer tissue (105–107). When EMT occurs, the main features of epithelial cells are lost, resulting in a change from polygonal to spindle-shaped fibroblast-like morphology. Additionally, the cells also lose their polarity, show reduced adhesion, and gain the ability to invade and metastasize (108, 109). EVs have a strong effect on OS invasive metastasis **(** **Table 2** **)**. When *in-vitro* synthesized miR-143 was transported into OS cells *via* EVs, they significantly inhibited the invasive ability of the cells (110). Gong et al. found that highly invasive OS cells secreted exosomal miR-675 into recipient cells and further suppressed CALN1 expression to enhance migration and invasion of OS cells. Additionally, serum exosomal miR-675 levels among OS cases are strongly associated with the prognosis of OS (111). Mazumdar et al. found that EVs derived from 143-B cells with high metastasis capacity and SAOS-2 cells with low metastasis capacity could induce the recruitment of BMCs into the lungs. The components of EVs might inhibit distant metastasis of OS (113). Zhong et al. showed that the Rab22a-NeoF1 fusion protein with PYK2 could be sorted into EVs in OS. The exosomal Rab22a-NeoF1 fusion protein promotes premetastatic lung niche generation by recruiting bone marrow-derived macrophages (BMDMs) (112). Han et al. showed that exosomal miR-1307 obtained from OS cells enhanced OS cell growth, invasion, and migration by inhibiting AGAP1 expression; thus, targeting miR-1307 might inhibit the malignant progression of OS (100).

**Table 2:** Biological functions of exosomes during the metastasis of OS.

EV content	Parent cells	Target cell	Mechanism	Biological activity	Ref.
synthetic miR-143	/	OS cells	/	Inhibit cell invasion	(110)
miR-675	OS cells	hFOB1.19	CALN1	Enhance OS cell invasion, and migration	(111)
Rab22a-NeoF1/PYK2	PYK2-positive osteosarcoma cells	macrophages	RhoA	Facilitate the pre-metastatic niche formation	(112)
miR-1307	OS cells	OS cells	AGAP1	Enhance OS cell growth, invasion, and migration	(100)

## EVs are essential for angiogenesis in osteosarcoma

Angiogenesis is the formation of new blood vessels in capillaries or venules behind capillaries (114, 115). This process is regulated by the interaction between proangiogenic and antiangiogenic factors. Although these factors are stable under normal physiological conditions, they can be activated or inactivated by external stimuli (12, 116). Different types of cells (cancer and healthy cells) require nutrients, which are supplied through blood capillaries. These capillaries can also excrete metabolic waste generated within cells (117, 118). Tumor-derived EVs are associated with an important mechanism that promotes angiogenesis. Moreover, EVs have a critical effect on angiogenesis in OS **(** **Table 3** **)**. Yoshida et al. found that the expression of miR-25–3p increased in OS tissues, which promoted cancer development, drug resistance, and invasion by inhibiting the expression of DKK3. Embedding synthetic miR-25–3p into tumor-derived EVs significantly promoted the capillary formation and vascular endothelial cell (EC) invasion (119). Tao et al. showed that angiogenesis in OS could be promoted by EWSAT1. Therefore, including exosomes increases the sensitivity of vascular endothelial cells, which directly induces an increase in the secretion of angiogenic factors (120). Li et al. showed that osteosarcoma cells with high exosome abundance could modulate autophagy and angiogenesis in OS *via* ATG and miR-153 by secreting exosomal lnc-OIP5-AS1 into other OS cells (121).

**Table 3:** The biological function of exosome in the angiogenesis of OS.

EV content	Parent cells	Target cells	Mechanism	Biological activity	Ref.
synthetic miR-25–3p	/	OS cells	DKK3	Enhance angiogenesis and vascular endothelial cell migration	(119)
EWSAT1	/	OS cells	/	Increase in sensitivity/reactivity of vascular endothelial cells	(120)
OIP5-AS1	OS cells	OS cells	miR-153/ATG5	Increase in the angiogenesis level	(121)

## Extracellular vesicles are essential for the immune activity of osteosarcoma

The natural response of the body to any foreign material is expressed by immune system activation and production of EVs (22, 83, 122). EVs can also regulate and modulate immune cells and participate in the immune response (21, 123, 124). EVs obtained from cancer cells can deliver tumor-associated antigens (TAAs) to stimulate immune cells and generate antitumor immune responses. However, they can also interfere with immune recognition and inhibit tissue-associated cells, T cells, immune-related cells, and natural killer (NK) cells, thus accelerating tumor cell escape and metastasis (25, 125). Moreover, EVs are responsible for regulating cancer cell development *via* TME-derived immune cells (126, 127). Additionally, the immune microenvironment within OS cells is strongly affected by EVs **(** **Table 4** **)**. Cancer-associated fibroblast (CAFs)-secreted exosomal miR-1228 can enhance OS migration and invasion *via* SCAI. This can be further used in the development of miR-1228-based anti-OS therapy (119). Raimondi et al. found that EVs can promote osteoclast bone resorption and differentiation. EVs can also enhance tube formation in ECs while increasing the expression of angiogenic markers. Specific miRNAs, including miR-21–5p and miR-148a, have important effects on the tumor microenvironment, as determined by second-generation sequencing (128). The EVs of metastatic OS cells secrete exosomal TGFβ2 into tumor-associated macrophages, which in turn promote the M2 phenotype and contribute to immunosuppression and tumorigenesis (129). Mazumdar et al. showed that EVs obtained from OS cells can promote the differentiation of myofibroblasts/CAF, the generation of fibronectin, and the expression of smooth muscle actin. They can also significantly promote the invasive ability of human lung fibroblasts (130). Cheng et al. showed that OS-obtained EVs can promote the polarization of M2 macrophages *via* Tim-3, which in turn can promote the invasion of OS cells and metastasis (135). Zhang et al. showed that OS cell-derived exosomal COL6A1 can convert normal fibroblasts into CAFs by secreting proinflammatory cytokines. After activation, CAFs can mediate the TGF-β/COL6A1 pathway to enhance the migration and invasion of OS cells (132). Zhang et al. showed that exosomal LIFR-AS1 obtained from macrophages could promote the OS malignancy grade by combining with miR-29a, which promoted the NFIA level (133).

**Table 4:** The biological functions of exosome in the immuno-modulation of OS.

EV content	Parent cell	Target cell	Mechanism	Biological function	Ref.
miR-1228	cancer-associated fibroblasts	OS cells	SCAI	Promote OS cell migration and invasion	(119)
miR-148a-3p and miR-21–5p	OS cells	Raw264.7 and Huvec cells	/	Influence osteoclast formation, tumor angiogenesis, and bone resorption	(128)
TGFβ2	Metastatic OS cells	Tumor-associated macrophages	/	Enhance M2 phenotype while creating the tumor-promoting, Immunosuppressive TME	(129)
TGFβ1	OS cells	Resident lung cells	/	Drive myofibroblast/cancer-associated	(130)
				fibroblast differentiation	
Tim-3	MG63	Macrophages	/	Induce M2 type differentiation of macrophages	(131)
COL6A1	OS cells	cancer-associated fibroblasts	IL-6, IL-8 and STAT1	Convert normal fibroblasts to cancer-associated fibroblasts	(132)
LIFR-AS1	Macrophages	OS cells	miR-29a/NFIA	Enhance OS cell growth, invasion, and migration	(133)
				While promoting their apoptosis	
miR-221–3p	M2-polarized tumor-associated macrophages	OS cells	SOCS3/JAK2/STAT3	Promote growth of OS cells	(134)

## Potential clinical application of EVs in osteosarcoma

Extracellular vesicles consist of various biomolecules, which are biologically active. They circulate through systemic circulation and are also found in various body fluids capable of mediating long-distance intercellular communication (40, 136). Tumor-derived EVs are rich in biomolecules, such as proteins, nucleotides, and lipids, which indicate the origin of the pathophysiological status of the cells (137, 138). EVs can provide a specialized lipid bilayer covering, thus preventing the degradation of RNA molecules (137, 139). Hence, the detection of tumor EVs in patients provides significant advantages to liquid biopsy, and EVs might also be used for early diagnosis. EVs might also be used to develop efficacious treatment strategies and monitor the prognosis of different diseases [181.182]. A specific collection of RNAs in the EV cargo might also serve as new or supplementary biomarkers in the diagnosis and progression of OS (31). Another study showed dysregulated levels of several miRNAs and mRNAs in EVs isolated from the serum of OS patients with a poor chemotherapeutic response compared to that of patients who responded positively to chemotherapy (140). A pilot study showed a higher tumor mutation burden in the RNA isolated from the plasma samples with metastatic EVs compared to that isolated from the plasma samples with non-metastatic ones (141). These findings highlighted the clinical application of EVs in OS.

## Extracellular vesicles are promising tools for developing osteosarcoma biomarkers

The diagnostic and prognostic assessment of OS improved considerably with the application of EVs as a biomarker for the disease. Next-generation sequencing was conducted, and eight novel miRNAs were identified from OS cells, out of which five miRNAs were present in circulating EVs among OS patients. However, the biological activity in the pathogenesis of OS and the diagnostic and therapeutic potential of these miRNAs need to be further investigated (142). The expression levels of plasma EV-miR-101 in OS patients and normal participants were determined by performing qRT–PCR. The results indicated a significant decrease in EV-miR-101 levels in OS patients relative to that in normal participants. Moreover, the EV-miR-101 plasma levels in OS patients with metastases were lower than those in patients without metastases. Hence, EV-miR-101 might be a diagnostic marker for OS (143). Ye et al. identified 57 differentially expressed miRNAs in plasma samples obtained from OS patients and normal participants *via* high-throughput sequencing. Among these miRNAs, 20 were upregulated, and 37 were downregulated. The expression of miR-92a-3p, miR-130a-3p, miR-195–3p, let-7i-3p, and miR-335–5p increased significantly within EVs from OS patients relative to their expression in controls. The findings suggested that these miRNAs might be used as potential diagnostic markers for OS (144). Zhang et al. reported high levels of CASC15 in OS cells and tissues along with a significant increase in the levels of CASC15 in the OS plasma EVs compared to their levels in controls (145). Cambier et al. described the significant diagnostic potential of overexpressed biomarkers such as HSATII, HSATI, Charlie 3, and LINE1-P1 at the DNA level rather than the RNA level in serum EVs from OS patients compared to their levels in serum EVs of the control (146). Huo et al. described significant upregulation of hsa_circ_0056285 in serum EVs in OS patients. They also showed the great diagnostic ability of hsa_circ_0056285 based on the ROC curve analysis (147). The expression of SENP1 obtained from plasma exosomes of OS patients was closely related to the tumor size, tumor location, necrosis rate, lung metastasis, and surgical staging. Moreover, patients with higher SENP1 expression had poorer overall survival, and disease-free survival (DFS) compared to OS patients with downregulated SENP1 (148). Han et al. analyzed EVs from plasma samples of OS patients with and without metastases and compared the results to those of normal controls using MALDI-TOF MS. They identified seven exosomal protein markers that were associated with OS lung metastasis (11). Also, noninvasive liquid biopsy using MALDI-TOF MS fingerprinting and SERS for the identification of EVs can be applied for the rapid diagnosis of OS (149).

## Potentials of EVs in the treatment of osteosarcoma

**Figure 2:**
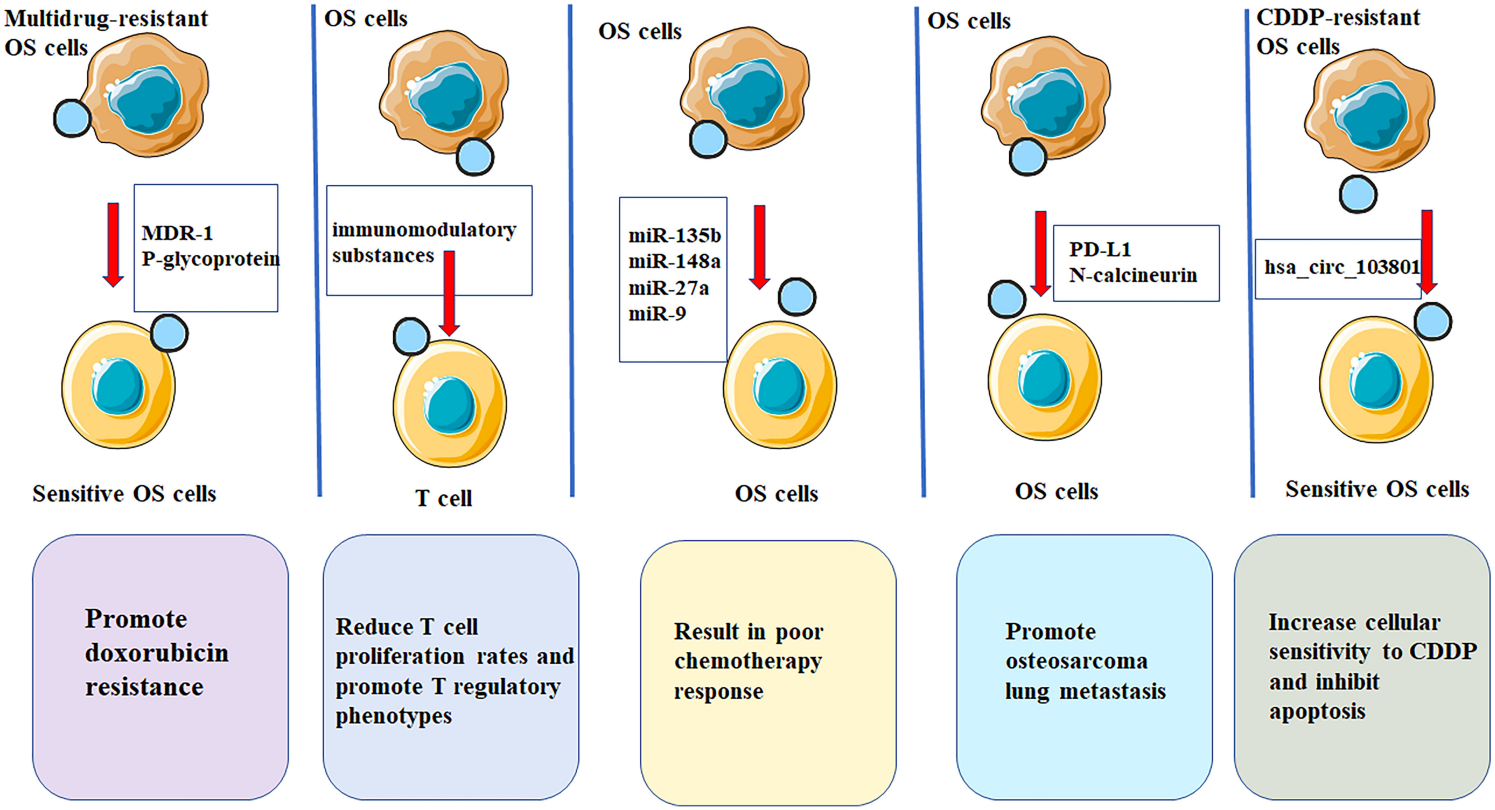
EVs have potential applications in treatment of OS. EVs are multifunctional nanostructured carriers which can be used as drug delivery systems with low immunogenicity as well as high biocompatibility and efficacy. OS-derived EVs contain immunomodulation properties that significantly reduces T cell proliferation rates and promote T regulatory phenotypes, thereby promoting OS progression. OS cases showing low chemosensitivity in patients showing favorable chemosensitivity. miR-9, miR-27a, miR-135b and miR-148a show marked up-regulation within serum EVs of OS patients. OS cells could promote osteosarcoma lung metastasis by releasing EVs that contained PD-L1 and N-calcineurin. EVs from cisplatin-resistant (CDDP)-resistant OS cells decreased P-glycoprotein and MDR-associated protein 1 levels in MG63 and U2OS cells, increases cellular sensitivity to CDDP and inhibits apoptosis through exosomal-hsa_circ_103801.

Treatment options for OS were either surgery or radiotherapy until the 1970s. Patients with OS also showed high resistance to radiotherapy (150, 151). Clinical results showed that surgical intervention, including tumor resection and/or amputation, cannot improve the survival rate (the operative mortality was about 80%) (152). The five-year survival rate of tumor resection cases is only 20% (153). Additionally, chemotherapeutic interventions can improve the survival rate of OS and reduce the amputation rate, thus improving the limb rescue score. The long-term survival rate of OS patients without metastasis is as high as 75%, compared to 20% before the 1970s (154, 155). However, the long-term survival rate of patients with recurrence or metastasis is still low **(** **Figure 2** **)**.

Kyung et al. showed that EVs have antitumor effects on osteosarcoma cells. EVs from canine macrophages can activate the apoptosis pathway of canine OS cells, which is an effective anti-cancer treatment (156). Additionally, MSC-derived EVs carrying miR-150 can reduce the proliferation and migration of osteosarcoma cells by targeting IGF2BP1 (insulin-like growth factor-2 mRNA binding protein 1) (157) Exosomes might also be used as a carrier to deliver chemotherapeutic drugs to osteosarcoma cells [188.189]. Exosomes can be directly charged with drugs [190.191].

## Conclusion

The advanced metastasized tumors, in contrast to primary tumors, often pose a major hindrance to the success of treatment outcomes in OS and increase patient mortality. Therefore, early diagnosis is the key to improving the prognosis and survival of OS patients (123, 158). EVs are stable, diverse, nano-sized vesicles that are found in most tissues, organs, and body fluids (124, 159). Moreover, EVs containing transmembrane proteins and some intracellular proteins, such as integrins or genetic material from the cells of origin, display a high level of identity within cells. This identity is associated with the identification of the tissue of origin, suggesting the importance of EVs and their potential as biomarkers in the early diagnosis and prognosis of OS (22, 160, 161). The surface proteins of EVs can be targeted and captured by recipient cells, and the contents of EVs can alter the physiological state of recipient cells (162, 163). Tumor EVs can also modulate cancer progression, immune evasion, metastasis, and angiogenesis by interacting with other cells within the TME (125, 164, 165). Additionally, the exosome-mediated pathological processes also highlight the great potential of EVs as biomarkers. Also, a better understanding of the mechanisms of exosome action is necessary to screen, diagnose, and assess patient prognosis.

There are still many problems in the development of EVs. For example, a standardized approach is needed for the quick, easy, and specific isolation of EVs in liquid biopsy. Moreover, EVs can serve as potential biomarkers for the diagnosis of OS, predict its prognosis, and monitor real-time treatment response. Clinical studies with a small sample size have shown reproducibility of EVs (166–168). However, more multicenter trials with large sample sizes are required for developing more accurate liquid biopsies. For evaluating the biological functions of EVs, determining whether they have similar regulatory functions *in vivo* and *in vitro* is challenging. The reason for this heterogeneity is that numerous assays have been performed *in vitro*, however, similar culture conditions cannot be replicated *in vivo*. Additionally, for therapeutic purposes, exosome-derived cells need to be selected carefully to ensure safe treatment. Due to their availability and non-nucleated and non-genetic nature, erythrocytes are the most promising cells for producing exosomes.

Besides their potential as good biomarkers, EVs are promising for precise and targeted cancer therapy (169–171). The development of a novel drug-loading system is a barrier to enhancing the effectiveness of antitumor drug therapy. Therefore, as a natural therapeutic carrier, EVs might be used for their low immunogenicity and various therapeutic bioactive molecules contained within (21, 161). Moreover, exogenous drugs carried by EVs can maintain drug stability *in vivo*. These advantages make EVs a better drug loading system than traditional drug delivery models. Hence, EVs are important for developing precision medicine for OS and other cancers. Han et al. constructed the iRGD-Lamp2b-modified MSC fusion gene for isolating and purifying EVs, as well as, loading the anti-miRNA-221 oligonucleotides into EVs. AMO-loaded EVs are effective in inhibiting *in-vitro* colon cancer (CC) cell growth and clone-forming ability (172). Exosomal ANXA6 levels in the sera of TNBC patients can predict the efficacy of gemcitabine chemotherapy (173). CC cells can produce exosomal miR-208b to receptor T-cells and promote the expansion of Treg cells *via* programmed cell death factor 4 (PDCD4), leading to malignant tumor growth and oxaliplatin resistance (174).

In this study, we highlighted and reviewed the advancements in the research on the biological functions of EVs during the occurrence and development of OS, along with its clinical applications. Moreover, EVs from OS can promote the progression of OS by regulating cancer drug resistance, immunity, angiogenesis, and metastasis. These findings highlight the role of EVs as anti-OS targets. Additionally, due to their abnormal expression in tumor-derived exosomal inclusions and their ability to reflect the tumor status, EVs might be used as markers for the diagnosis and prognosis of OS. Exosomal drug carriers and immunomodulatory therapy are promising therapeutic strategies in the treatment of OS.

## Author contributions

SL, XG, and BG were responsible for original drafting, supplementation, allocation as well as editing. The authors have carefully read and approved the final version for submission.

## Funding

The present study was funded by Fundamental Research Funds for the Central Universities (LD202110) and Natural Science Foundation of Liaoning Province (2020-MS-058) and Shenyang Young and Middle-age Scientific and Technological Innovation Talent Support Plan (RC190456).

## Acknowledgments

The present study was funded by Liaoning Cancer Hospital & Institute (Shenyang) and China Medical University (Shenyang).

## Conflict of interest

The authors declare that the research was conducted in the absence of any commercial or financial relationships that could be construed as a potential conflict of interest.

## Publisher’s note

All claims expressed in this article are solely those of the authors and do not necessarily represent those of their affiliated organizations, or those of the publisher, the editors and the reviewers. Any product that may be evaluated in this article, or claim that may be made by its manufacturer, is not guaranteed or endorsed by the publisher.

## Abbreviations

OS, Osteosarcoma; ALP, alkaline phosphatase; LDH, lactate dehydrogenase; lncRNAs, long non-coding RNAs; miRNAs, microRNAs; mRNAs, messenger RNAs; circRNAs, circular RNAs; ASMCs, airway smooth muscle cells; NSCLC, non-small-cell lung cancer; LUAD lung adenocarcinoma; EVs, extracellular vesicles, ILVs, luminal vesicles; MVBs, multivesicular bodies; Aiix, ALG-2 interacting protein X; ESCRT, endosomal sorting complex required for transport; HGG, high-grade gliomas; PSA, prostate-specific antigen; EMT, epithelial-mesenchymal transition; MSCs, mesenchymal stem cell; CAFs, cancer-associated fibroblasts; BMSCs, bone marrow-derived mesenchymal stem cells; OS, overall survival; DFS, disease-free survival; NGS, next-generation sequencing; CDDP, cisplatin-resistant.
